# Effects of Varying Glucose Concentrations on ACE2′s Hypothalamic Expression and Its Potential Relation to COVID-19-Associated Neurological Dysfunction

**DOI:** 10.3390/ijms23179645

**Published:** 2022-08-25

**Authors:** Ankita Srivastava, Bashair M. Mussa

**Affiliations:** 1 Sharjah Institute for Medical Research, College of Medicine, University of Sharjah, Sharjah 27272, United Arab Emirates; 2 Basic Medical Science Department, College of Medicine, University of Sharjah, Sharjah 27272, United Arab Emirates

**Keywords:** COVID-19, SARS-CoV-2, ACE2, diabetes mellitus, neurological impairment, hypothalamus

## Abstract

The coronavirus disease 2019 (COVID-19) pandemic has negatively impacted millions of lives, despite several vaccine interventions and strict precautionary measures. The main causative organism of this disease is the severe acute respiratory syndrome-coronavirus-2 (SARS-CoV-2) which infects the host via two key players: the angiotensin-converting enzyme 2 (ACE2) and the transmembrane protease, serine 2 (TMPRSS2). Some reports revealed that patients with glycemic dysregulation could have increased susceptibility to developing COVID-19 and its related neurological complications. However, no previous studies have looked at the involvement of these key molecules within the hypothalamus, which is the central regulator of glucose in the brain. By exposing embryonic mouse hypothalamic neurons to varying glucose concentrations, we aimed to investigate the expression of ACE2 and TMPRSS2 using quantitative real time polymerase chain reaction and western blotting. A significant and time-dependent increase and decrease was observed on the viability of hypothalamic neurons with increasing and decreasing glucose concentrations, respectively (*p* < 0.01 and *p* < 0.001, respectively). Under the same increasing and decreasing glucose conditions, the expression of hypothalamic ACE2 also revealed a significant and time-dependent increase (*p* < 0.01). These findings suggest that SARS-CoV-2 invades the hypothalamic circuitry. In addition, it highlights the importance of strict glycemic control for COVID-19 in diabetic patients.

## 1. Introduction

Ever since the first reported case in late December of 2019 in Wuhan, China, there has been much speculation regarding the nature of the severe acute respiratory syndrome-coronavirus-2 (SARS-CoV-2), the causative organism of coronavirus disease 2019 (COVID-19). Several studies have elucidated the pathogenesis of COVID-19 and the associated respiratory, cardiovascular, metabolic, and neurological symptoms. In addition, studies have identified the multiple risk factors of COVID-19, and these include old age, hypertension, obesity, and metabolic disorders such as diabetes mellitus (DM) [1,2,3,4,5,6]. Interestingly, previous studies that have examined the association between DM and COVID-19 severity have pointed towards a higher percentage of DM patients (19%) developing acute respiratory distress syndrome (ARDS). This complication could be due to an excessive, uncontrolled, and dysregulated inflammatory response to SARS-CoV-2 in this group of patients [3,7,8].

Wu et al.’s study conducted on 201 patients demonstrated that hyperglycemia was one of the main risk factors in the development of ARDS in patients with COVID-19 [8]. In another study, Yang et al. observed hyperglycemia as an independent predictor of death in the SARS epidemic [9]. Based on these findings, we postulate that glucose dysregulation could induce a cytokine storm resulting in the breakdown of the blood–brain barrier (BBB), leading to neurological impairment in patients with DM. Similarly, hypoglycemia or low blood glucose concentrations could also result in adverse outcomes in this group of patients. Given its complex pathogenesis, it is of great interest to study the factors influencing COVID-19 susceptibility and the associated neurological impairment in DM and understand the interrelationship between Angiotensin Converting Enzyme 2 (ACE2), DM, and COVID-19 as highlighted by Pal et al. [10]. 

Recently, our group proposed possible routes of entry of the SARS-CoV-2 virus into the brain [11]. Another study by our group also revealed potential microRNAs that could regulate this mechanism [12]. A recent report by Mao et al. further highlighted the neuro-invasive potential of SARS-CoV-2, where they found that 1 in 3 severe COVID-19 cases had CNS involvement [13,14]. The critical molecule facilitating this could be ACE2. Viruses such as SARS-CoV-2 can efficiently utilize this molecule to enter host cells to cause subsequent infection [15].

Few studies have documented the presence of ACE2 in the paraventricular nucleus (PVN) and supramammillary nuclei of the hypothalamus [13,16,17,18]. Recently, Hernandez et al. highlighted the possible contributions of these nuclei and their circuity in the development of COVID-19 related CNS disease [18]. Despite this, most studies investigated the encephalic expression of ACE2 in the cortex, which has overshadowed other vital regions that might be involved in CNS impairment and infection, such as the hypothalamus. 

The complex circuitry of the hypothalamus regulates several critical physiological and neuroendocrine responses such as thermo- and cardiovascular-regulation, respiration, stress, consciousness, gonadal hormone production, and the regulation of blood glucose [19,20,21,22,23]. Interestingly, most of these functions also govern the risk factors commonly associated with COVID-19 [4,24,25,26]. Despite the recent surge in COVID-19 cases that present with neurological symptoms such as headache, unconsciousness, nausea, brain fog, loss of olfaction and gustation, and encephalitis, little is known about the exact pathogenesis of SARS-CoV-2 in causing subsequent neurological impairment [27]. A preprint by Nampoothiri et al. (bioRxiv 2020.06.08.139329) gives a detailed review of the hypothalamus acting as a potential hub for SARS-CoV-2 infection [28]. However, no study has investigated the relationship between poor glycemic control and its consequences in causing COVID-induced neurological impairment with a specific focus on ACE2 in the hypothalamus. 

In addition to ACE2, SARS-CoV-2 also requires cellular proteases to enter the host cells efficiently. The most utilized is the transmembrane protease, serine two encoded by the TMPRSS2 gene. Viruses such as SARS-CoV-2 can alter the expression of TMPRSS2 in bronchial epithelial cells, emphasizing its pivotal role in viral entry [29,30]. Although some studies have determined the expression of TMPRSS2 in neuroblastoma, microglial and olfactory cells, its expression in hypothalamic cells remains unclear [31,32]. 

Despite this evidence, there is a lack of knowledge about the exact pathogenesis of SARS-CoV-2 in causing COVID-19-related neurological impairment in patients with glycemic dysregulation. Therefore, considering these ambiguities, the objective of the present study was to investigate the expression of ACE2 and TMPRSS2 in response to varying glucose concentrations using embryonic mouse hypothalamic neurons as a research model. 

## 2. Results

The effects of various glucose concentrations on cell viability, gene, and protein expression of hypothalamic ACE2 and TMPRSS2 were investigated in two different groups of experiments. In the first group, increased concentrations of glucose (group 1:5400, 10,800, 16,200, and 21,600 mg/L) were used at different time points (24, 48 and 72 h), and in the second group, decreased concentrations of glucose were used (group 2: 2000, 900, 500, 200 mg/L;) at the same time points (24, 48, 72 h). Glucose concentration of 4500 mg/L was considered as the control group.

### 2.1. Investigating Cell Viability of Hypothalamic Neurons under Various Glucose Concentrations at Different Time Points

**Figure 1:**
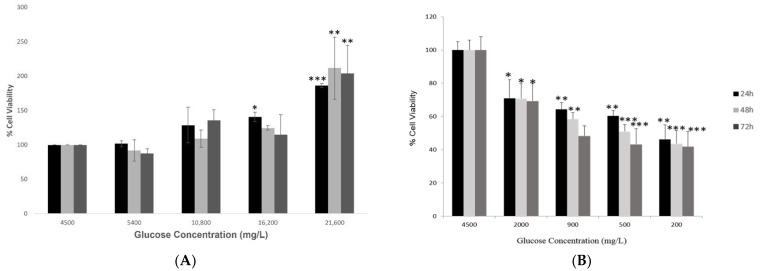
Cell viability of hypothalamic neurons under various glucose concentrations at different time points. (**A**) Increased glucose concentrations (mg/L) of 5400, 10,800, 16,200, and 21,600 (please check) at 24, 48 and 72 h time points enhanced the viability of cells significantly, with maximum percentage (~200%) observed at the highest glucose concentration of 21,600 mg/L compared to the control condition (4500 mg/L). (**B**) Low glucose concentrations (mg/L) of 2000, 900, 500, and 200 at 24, 48 and 72 h time points affected the viability of cells adversely, with a maximum reduction observed at the lowest glucose concentration of 200 mg/L at longest exposure of 72 h as compared to the control condition (4500 mg/L). The effects of decreasing glucose concentrations on the viability of hypothalamic neurons were published previously by our group. Data is represented as mean ± SEM (*n* = 3, * *p* < 0.05, ** *p* < 0.01, *** *p* < 0.001).

The investigation of cell viability of hypothalamic neurons under increased glucose concentrations (group 1) at different time points (24, 48 and 72 h) showed that an increase in glucose levels enhanced the survival of the hypothalamic neurons. At the highest glucose concentration of 21,600 mg/L, there was a significant increase (*p* < 0.01) as compared to the control condition of 4500 mg/L glucose at all three time points (Figure 1A).

However, treating hypothalamic neurons with low glucose concentrations (group 2) for 24, 48 and 72 h showed a significant and dose-dependent decrease in cell viability (Figure 1B). This effect was duration-dependent, and as the duration of exposure to low glucose concentrations increased, the cell viability reduced significantly compared to the control condition. This result was adapted from our study published previously [33].

### 2.2. Effect of Various Glucose Concentrations on Gene Expression of Hypothalamic ACE2 at Different Time Points

**Figure 2:**
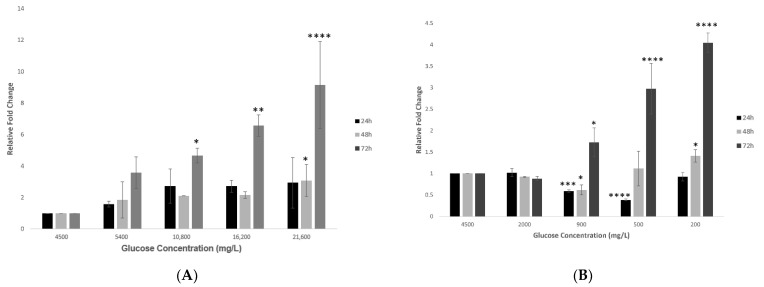
Gene expression of *ACE2* in hypothalamic neurons under various glucose concentrations at different time points. (**A**) Increase in glucose concentrations (mg/L) of 5400, 10,800, 16,200, and 21600 at 24, 48 and 72 h time points showed an increase in *ACE2*′s gene expression, with significant fold changes observed at the higher concentrations of 10,800, 16,200, and 21,600 mg/L after longest exposure of 72 h as compared to the control condition (4500 mg/L). (**B**) Decreasing glucose concentrations (mg/L) of 2000, 900, 500, and 200 at 24, 48 and 72 h time points also showed an increase in gene expression of *ACE2*, with significant fold changes observed at the lower concentrations of 500 and 200 mg/L at 72 h time point, as compared to the control condition (4500 mg/L). Data is represented as mean ± SEM (*n* = 4, * *p* < 0.05, ** *p* < 0.01, *** *p* < 0.001, **** *p* < 0.0001).

A significant increase in the gene expression of hypothalamic *ACE2* was observed in response to increasing concentrations of glucose (group 1) at different time points (24, 48 and 72 h). The most significant increase (*p* < 0.0001) in the gene expression of hypothalamic *ACE2* was reported at the most prolonged exposure of 72 h, as compared to the control condition of 4500 mg/L (Figure 2A). 

Treating hypothalamic cells with decreasing glucose concentrations (group 2) after 24 h showed an initial and significant reduction in *ACE2* expression at the 900 and 500 mg/L glucose concentrations, which then increased at the lowest glucose concentration of 200 mg/L (Figure 2B). On the other hand, after a longer exposure of 48 h, a dose-dependent increase in the expression of the *ACE2* gene became evident. This effect was significant after 72 h for the two lowest glucose concentrations of 500 and 200 mg/L (*p* < 0.01 and *p* < 0.0001 respectively; Figure 2B).

### 2.3. Effect of Various Glucose Concentrations on Protein Expression of Hypothalamic ACE2 at Different Time Points

**Figure 3:**
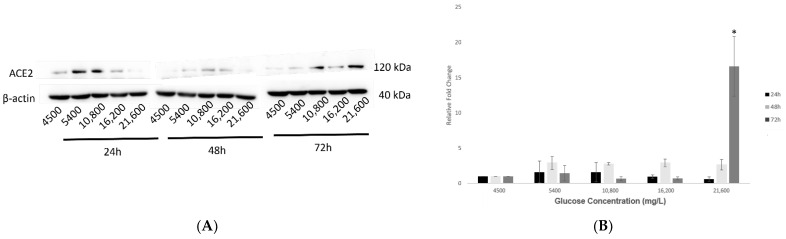

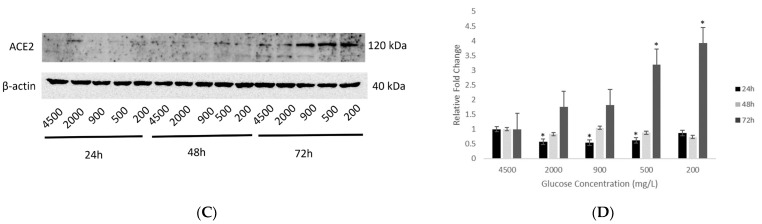
Protein expression of ACE2 in hypothalamic neurons under various glucose concentrations at different time points. (**A**) With increasing glucose concentrations (mg/L) of 5400, 10,800, 16,200, and 21,600, ACE2 (120 kDa) showed an upward trend in protein expression, with significant upregulation (*p* < 0.05) observed at the highest concentration of 21,600 mg/L after 72 h exposure as compared to the control condition (4500 mg/L). (**B**) Histogram with relative fold change for ACE2′s protein expression (*n* = 3) with increasing glucose concentrations compared to loading control (β-actin; 40 kDa). (**C**) With decreasing glucose concentrations (mg/L) of 2000, 900, 500, and 200, ACE2′s protein expression increased significantly (*p* < 0.05) at longest exposure of 72 h and at the lowest concentrations of 500 and 200 mg/L glucose, as compared to the control condition (4500 mg/L). (**D**) Histogram with relative fold change for ACE2′s protein expression (*n* = 3) with decreasing glucose concentrations compared to loading control (β-actin). Data is represented as mean ± SEM (* *p* < 0.05). Full blot images are provided in Supplementary Materials (Figures S1 and S2).

The analysis of corresponding protein expression of ACE2 in response to increased glucose concentrations (group 1) has revealed similar outcomes as its gene expression, where there was a dose- and time-dependent increase. The most significant expression (*p* < 0.05) was noted at the highest concentration of 21,600 mg/L glucose and after the most prolonged exposure of 72 h, as shown in Figure 3A,B, respectively.

Similarly, treatments with reduced glucose concentrations (group 2) at 24, 48 and 72 h showed that longer exposures at 48 and 72 h upregulated ACE2 protein expression, with the most significant enhancement (*p* < 0.05) noted at the lowest concentrations of 500 and 200 mg/L glucose (Figure 3C,D). 

### 2.4. Effect of Various Glucose Concentrations on Gene Expression of Hypothalamic TMPRSS2 at Different Time Points

**Figure 4:**
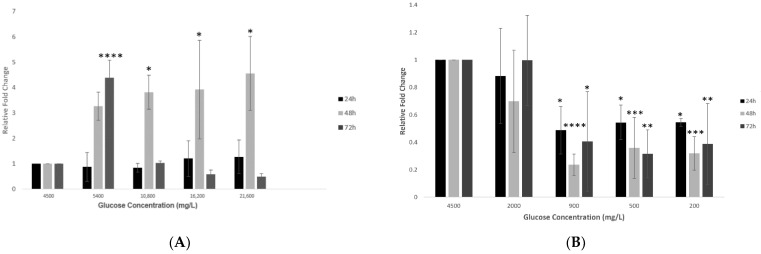
Gene expression of *TMPRSS2* in hypothalamic neurons under various glucose concentrations at different time points. (**A**) Increase in glucose concentrations (mg/L) of 5400, 10,800, 16,200, and 21,600 at 24, 48 and 72 h time points showed that *TMPRSS2* expression increased slightly at 24 h and significantly after 48 h but decreased after 72 h, as compared to the control condition (4500 mg/L). (**B**) With decreasing glucose concentrations (mg/L) of 2000, 900, 500, and 200, at the lowest concentrations of 900, 500, and 200 mg/L, *TMPRSS2* expression was observed to decrease significantly compared to the control condition (4500 mg/L). Data is represented as mean ± SEM (*n* = 4, * *p* < 0.05, ** *p* < 0.01, *** *p* < 0.001, **** *p* < 0.0001).

Treating hypothalamic neurons with increased glucose concentrations (group 1) revealed a dose-dependent increase in the gene expression of *TMPRSS2* at the 24 h time point (Figure 4A). This increase was significantly enhanced after 48 h, while after 72 h, *TMPRSS2* expression first increased significantly at 5400 mg/L and then decreased with increasing glucose concentrations (*p* < 0.0001 and *p* < 0.05 respectively; Figure 4A).

However, the opposite effect was observed with decreasing glucose concentrations (group 2), where the overall *TMPRSS2* expression significantly reduced (*p* < 0.05 to *p* < 0.001) at lower glucose concentrations, but this effect was not consistent across the three time points (Figure 4B). The opposite effect was observed with decreasing glucose concentrations (group 2), where the overall *TMPRSS2* expression significantly reduced (*p* < 0.05 to *p* < 0.001) at lower glucose concentrations, but this effect was not consistent across the three time points (Figure 4B). 

### 2.5. Effect of Various Glucose Concentrations on Protein Expression of Hypothalamic TMPRSS2 at Different Time Points

**Figure 5:**
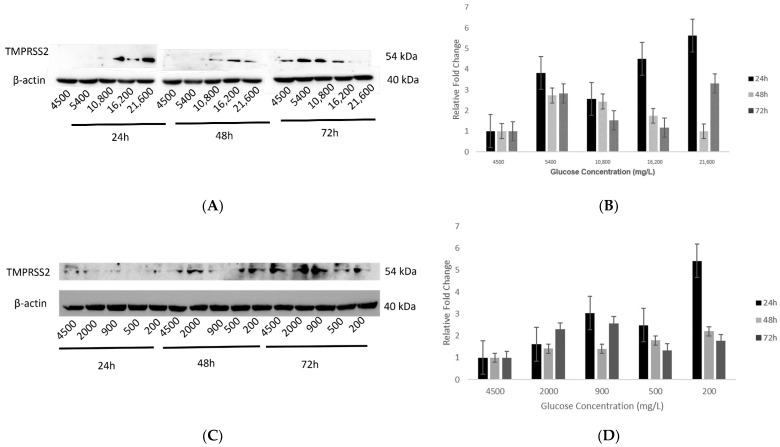
Protein expression of TMPRSS2 in hypothalamic neurons under various concentrations of glucose at different time points. (**A**) With increasing glucose concentrations (mg/L) of 5400, 10,800, 16,200, and 21,600, protein expression of TMPRSS2 (54 kDa) showed an increase after 24h, which with longer exposures of 48 and 72 h, decreased as compared to the control condition (4500 mg/L) which was not significant. (**B**) Histogram with relative fold change for TMPRSS2′s protein expression (*n* = 3) with increasing glucose concentrations compared to loading control (β-actin; 40 kDa). (**C**) With decreasing glucose concentrations (mg/L) of 2000, 900, 500, and 200, there was an increase in TMPRSS2′s protein expression after 24 h, whereas the opposite trend was observed after 48 h. However, after more prolonged exposure to 72 h, its expression first increased (at 2000 and 900 mg/L) and then decreased (at 500 and 200 mg/L concentrations), as compared to the control condition (4500 mg/L) but was not significant. (**D**) Histogram with relative fold change for TMPRSS2′s protein expression (*n* = 3) with decreasing glucose concentrations compared to loading control (β-actin). Data are represented as mean ± SEM. Full blot images are provided in Supplementary Materials (Figures S3 and S4).

Expression of hypothalamic TMPRSS2 increased in response to increasing glucose concentrations (group 1) at the 24 h time point. However, an opposite effect was observed after 48 h, and an inconsistent effect was observed after 72 h of exposure (Figure 5A,B). 

Effect of decreasing glucose concentrations (group 2) on protein expression of TMPRSS2 showed a slight increase at the 24 h time point whereas, for the 48 h time point, this increase was not significant (Figure 5C,D). Similarly, after 72 h of exposure, TMPRSS2 expression first increased at the 2000 and 900 mg/L glucose concentrations, but at the lower concentrations of 500 and 200 mg/L, there was an insignificant decrease (Figure 5C,D).

## 3. Discussion

Although SARS-CoV-2 is deemed a highly contagious respiratory coronavirus, its exact pathogenesis remains unknown. SARS-CoV-2 can infect some individuals more severely than others, given the risk factors of old age and pre-existing conditions such as DM. These predispositions have contributed significantly to its stature as a causative agent of a pandemic causing millions of deaths worldwide along with long-term and post-COVID-19 neurological effects [34]. We and others have elucidated the main routes by which COVID-19 can enter the brain and cause encephalitis, stroke, or lack of oxygen [11,35]. Several other studies have reported shreds of evidence of such damage in the autopsied brains of patients that did not experience any neurological symptoms while they were alive [36]. Budson et al.’s study also revealed a significant decline in cognitive, behavioral, and psychological function in hospitalized patients due to severe COVID-19. The outcomes of this study suggested several pathological mechanisms, including silent hypoxia and inflammatory responses as the underlying causes of this decline [34]. Together, these pieces of evidence prove the existence of “long-term COVID” that could also increase the risk of developing other neurological disorders in the future [37,38,39]. 

We conducted in vitro experiments using mouse embryonic hypothalamic cells under varying glucose conditions in the present study to correlate the effects of glycemic dysregulation and COVID-19-associated neurological impairment. In the first set of experiments, the viability of hypothalamic neurons was assessed, and the optimal glucose concentrations were determined. Under conditions of increasing glucose, the cell viability enhanced exponentially with increased exposure times. Physiologically, this enhanced viability of neurons is understandable. There would be an increased demand for the synthesis of nucleic acids and lipids, which are the building blocks of the embryonic brain and are essential for neuronal processes such as cell proliferation and synaptogenesis [40]. Ito et al. noticed a similar outcome in human pancreatic epithelial cells [41]. Incubating hypothalamic neurons with decreasing glucose concentrations significantly reduced their viability with prolonged exposures of 48 and 72 h. This observation is expected as glucose is vital for cellular metabolic processes, and the absence of significant glucose can lead to neuronal apoptosis [33]. Interestingly, at the shortest duration of 24 h, an opposite response occurred at the 900 and 500 mg/L concentrations, showing a decrease in *ACE2* gene expression. This could be attributed to an episodic change corresponding to a sudden drop in glucose concentration. It is very fascinating that reducing the concentration further to 200 mg/L increased *ACE2*′s gene expression, which supports our hypothesis linking decreased glucose with increased *ACE2* expression.

Arguably, longer exposure to varying glucose concentrations could be considered as a more appropriate in vitro research model that mimics DM pathologically. However, considering cell viability as an essential aspect of the present study, 72 h was chosen as the longest incubation time as substantial cell loss (>50%) occurred beyond this time point under decreasing glucose concentrations. 

It was essential to investigate the role of the key facilitators of SARS-CoV-2: ACE2 and TMPRSS2, to understand the ability of this virus to attack the hypothalamus. ACE2 is a well-known regulator of the renin-angiotensin aldosterone system (RAAS) that regulates cardiovascular functions [42]. Under normal physiological conditions, the expression of ACE2 in the hypothalamus is low [43]. However, results from the present study reveal that prolonged exposure to varying glucose concentrations caused a significant upregulation in the expression of hypothalamic ACE2. In agreement with these findings, previous research has observed the amplification of ACE2 under conditions of stress and inflammation [7,44]. In our study, exposing hypothalamic neurons to stress conditions induced by varying glucose concentrations could explain the upregulation of ACE2. Notably, an apparent increase in cellular ACE2 expression could increase the binding efficiency of the SARS-CoV-2 receptors to the host cell. This increase in binding may result in a higher possibility of an inflammatory cytokine storm that is known to be associated with increased severity of the disease [45,46]. However, the involvement of other pro- and anti-inflammatory molecules of the RAAS system in COVID-19 can be tricky. While the canonical ACE/Ang-II/AT1R axis can cause a proinflammatory response and decrease endothelial permeability, the secondary, non-canonical axis can counter-regulate this response to produce anti-inflammatory outcomes [47]. Therefore, further research is imperative to understand the exact roles of these molecules in COVID-19.

In contrast, for TMPRSS2, a priming protease facilitating entry of SARS-CoV-2 into host cells, the outcomes of gene and protein expression analyses were not conclusive. Especially, a significant increase in its gene expression at 5400 mg/L glucose was uncanny. Perhaps, this concentration is most favorable for TMPRSS2 within the hypothalamus. However, given the differential expression of TMPRSS2 in the brain, where it is found only in the cortex, cerebellum, and hypophysis and the lack of knowledge about its inherent expression in the hypothalamus makes it challenging to explain the differential outcomes obtained in the present study [31]. However, therapeutic interventions are also being designed for TMPRSS2 in fighting COVID-19, which makes it a potential target for further investigation [48,49].

### Limitations of the Study

Using an immortalized cell line as a research model gave us the advantages of easy culturing, higher experimental reproducibility, and preserved neuronal phenotypes [50]. Perhaps, using primary neuronal cells would have been a better choice, but since mature neuronal cells cannot divide, culturing these cells represents a major challenge. Furthermore, the use of human hypothalamic cells would provide a better representation to study these interactions, but given several challenges associated with using human cell lines, using a mouse model for in vitro research was the best available option.

Nevertheless, one of the main limitations of this study is the use of only one cell line. Therefore, future directions would involve replicating the outcomes of this study in other embryonic- and adult-hypothalamic neurons to deepen the understanding of the pathophysiology of SARS-CoV-2 infecting the hypothalamus. Moreover, in vitro studies alone are inadequate to support the involvement of hypothalamic neurons in the pathophysiology of diabetes-related COVID-19 susceptibility. Therefore, using network analysis to investigate differentially expressed and correlated genes between DM and COVID-19 could provide further insights into the development of common therapeutic targets for this group of patients and highlight the neuro-invasive potential of the SARS-CoV-2 virus. This investigation could help enforce changes in the current treatment course centered only around respiratory and cardiovascular complications.

## 4. Materials and Methods

### 4.1. Cell Culture and Maintenance

Embryonic mouse hypothalamic cells (mHypoE-N39; Cedarlane, Burlington, ON, Canada) were grown in Dulbecco’s Modified Eagles Medium (DMEM 6429; Sigma Aldrich, St. Louis, MO, USA) supplemented with 10% fetal bovine serum (Sigma Aldrich, St. Louis, MO, USA) and 1% penicillin-streptomycin (Sigma Aldrich, St. Louis, MO, USA) and maintained in a humidified atmosphere at 37 °C with 5% CO_2_. Cells were cultured under increased glucose wherein an additional amount of 1M D-glucose (Sigma Aldrich, St. Louis, MO, USA) was added to DMEM 6429 to achieve concentrations of 5400, 10,800, 16,200, and 21,600 mg/L glucose. Additionally, cells were cultured under reduced glucose concentrations of 2000, 900, 500, and 200 mg/L using DMEM 6429 with 500 mg/L glucose and DMEM without (w/o) glucose (bioWORLD, Dublin, OH, USA) media. The recipes for increasing and decreasing glucose concentrations are provided in Table S1 (Supplementary Materials). 

### 4.2. Cell Viability

Approximately 10,000 cells were seeded per well in 96-well plates (Jet Biofil, Guangzhou, China) with 200 μL of DMEM 6429 and maintained in the incubator at 37 °C with 5% CO_2_. Upon reaching >90% confluency, cells were treated in triplicates with glucose (groups 1 and 2) for 24, 48, and 72 h. Post incubation, cell viability was assessed using a colorimetric assay with the tetrazolium dye MTT (3-[4,5-dimethylthiazol-2-yl]-2,5 diphenyltetrazolium bromide; Sigma Aldrich, St. Louis, MO, USA). Culture medium was discarded and replaced with a mixture containing 20 μL of MTT (5 mg/mL) dissolved in 100 μL of PBS (Phosphate Buffer Saline; Sigma Aldrich, St. Louis, MO, USA). Cells were then incubated at 37 °C for 2 h in the dark. After this incubation, 100 μL of dimethyl sulfoxide (DMSO; Sigma Aldrich, St. Louis, MO, USA) was added to each well to dissolve the formazan crystals formed by MTT. Absorbance was recorded at 570 nm on a microplate reader (Agilent BioTek, Winooski, VT, USA). The percentage of cell viability was calculated as per the following equation: % cell viability = (OD of sample at 570 nm/OD of control at 570 nm) × 100.

### 4.3. Gene Expression

Upon reaching 80% confluency, approximately 2.5 × 105 cells were seeded in 6-well plates (Corning, NY, USA) and maintained under increasing and decreasing glucose concentrations (Table S1) for 24, 48 and 72 h. RNA was isolated from the cells using the easy-BLUE Total RNA Extraction Kit (iNtRON Biotechnology Inc., Burlington, MA, USA) as per the manufacturer’s instructions. Eluted RNA was then reverse-transcribed to 1000 ng of cDNA using the High-capacity cDNA synthesis kit (Applied Biosystems, Foster City, CA, USA). A measure of 1µL of cDNA was used from the latter to perform quantitative PCR using the PowerUp SYBR green master mix (Thermo Fisher Scientific, Waltham, MA, USA) in a total reaction volume of 10 µL, containing 5 µL of the master mix, 1 µL of 10 µM forward primer, 1 µL of 10 µM reverse primer and 2 µL of nuclease-free water per reaction. The reactions were set up in triplicate, and the experiment was performed three times for reproducibility using the QuantStudio 3 Real-Time PCR system (Applied Biosystems, Foster City, CA, USA). Cycling parameters were followed as per the manufacturer’s protocol for a standard reaction. The primer sequences used for the housekeeping gene—Glyceraldehyde 3-phosphate dehydrogenase (GAPDH), ACE2, and TMPRSS2 are listed in Table S2 (Supplementary Materials).

### 4.4. Protein Expression

Approximately 1 × 106 cells were seeded in 100mm dishes (Corning, NY, USA). They were exposed to increasing and decreasing glucose concentrations for 24, 48 and 72 h. Then, the protein was isolated using the M-PER Mammalian Protein Extraction Reagent (Thermo Fisher Scientific, Waltham, MA, USA) as per the manufacturer’s instructions. Isolated protein was then quantified using the Bradford reagent (BioRad Laboratories, Hercules, CA, USA), and 40 µg of protein from each sample was run on a 10% SDS-PAGE gel. The gel was then transferred onto a nitrocellulose membrane (BioRad Laboratories, Hercules, CA, USA) and blocked in 5% skim milk powder (Sigma Aldrich, St. Louis, MO, USA) for 1 h at room temperature (RT). Following blocking, the blots were incubated with primary antibodies against ACE2 (1:1000; A12737; Abclonal, Woburn, MA, USA), TMPRSS2 (1:1000; ab214462; Abcam, Cambridge, UK), and the loading control of anti-beta-actin (1:2000; Sigma Aldrich, St. Louis, MO, USA). On the following day, blots were washed and incubated with the appropriate secondary antibodies (anti-mouse IgG [7076S] and anti-rabbit IgG [7074S]; 1:1000; Cell Signaling Technology, Danvers, MA, USA) before visualization using Clarity Western ECL substrate reagent (BioRad Laboratories, Hercules, CA, USA) in a BioRad chemi-doc imaging system (BioRad Laboratories, Hercules, CA, USA). 

### 4.5. Statistical Analysis

DMEM 6429 with 4500 mg/L glucose was used as the control condition for statistical analyses. Data were expressed as mean ± SEM. Statistical analyses were performed using the GraphPad Prism 9 software for Windows (GraphPad Software, CA, USA) using ordinary one-way ANOVA followed by Tukey’s correction multiple comparisons. For protein expression, Western blot images were quantified using the ImageJ software (version 1.8, NIH, USA) and Student’s *t*-test was used to calculate the significance. *P*-values less than 0.05 were considered significant. 

## 5. Conclusions

One of the main findings of the present study is the significant increase in both the mRNA and protein expression of ACE2 with varying glucose concentrations. In addition, this expression was highest in response to prolonged exposure to the treatments. The outcomes of this study may help in explaining why patients with DM are more susceptible to COVID-19-related neurological impairment. Furthermore, they will help identify novel potential therapeutic targets and highlight the importance of obtaining strict glycemic control in this vulnerable group. 

## Data Availability

Not applicable.

## Supplementary Materials

The supporting information can be downloaded at: https://www.mdpi.com/article/10.3390/ijms23179645/s1.
