# Supplementary material for: Effects of Varying Glucose Concentrations on ACE2′s Hypothalamic Expression and Its Potential Relation to COVID-19-Associated Neurological Dysfunction
Source: Int J Mol Sci. 2022 Aug 25;23(17):9645. doi: 10.3390/ijms23179645 (PMC9455961; doi:10.3390/ijms23179645)
Supplement: Supplementary file 1 [file ijms-23-09645-s001.zip › ijms-1814903-Supplementary.pdf]

Supplementary Material

Table S1. Recipes for increasing and decreasing glucose concentrations using DMEM with 4500 mg/L glucose, DMEM w/o glucose, and 1M D-glucose for a total volume of 50ml per treatment condition.

Glucose Concentration (mg/L)	DMEM with 4500 mg/L glucose (mL)	DMEM w/o Glucose (mL)	1M D-Glucose (µL)
**Decreasing:**			
2000	22.2	27.8	-
900	10	40	-
500	5.6	44.4	-
200	2.22	47.78	-
**Increasing:**			
5400 **(~30mM)**	49.75	-	250
10800 **(~60mM)**	48.25	-	1750
16200 **(~90mM)**	46.75	-	3250
21600 **(~120mM)**	45.25	-	4759

Table S2. Mouse primer pairs used for quantitative real-time polymerase chain reaction.

Gene	Forward Primer (5′–3′)	Reverse Primer (5′–3′)	Accession No.
**ACE2**	TCCATTGGTCTTCTGCCATCC	AACGATCTCCCGCTTCATCTC	NM_027286
**TMPRSS2**	GAGAACCGTTGTGTTCGTCTC	GCTCTGGTCTGGTATCCCTTG	NM_015775
**GAPDH**	GAAATCCCATCACCATCTTCCAGG	GAGCCCCAGCCTTCTCCATG	NM_002046

Western Blot Images (without editing):

Figure S1

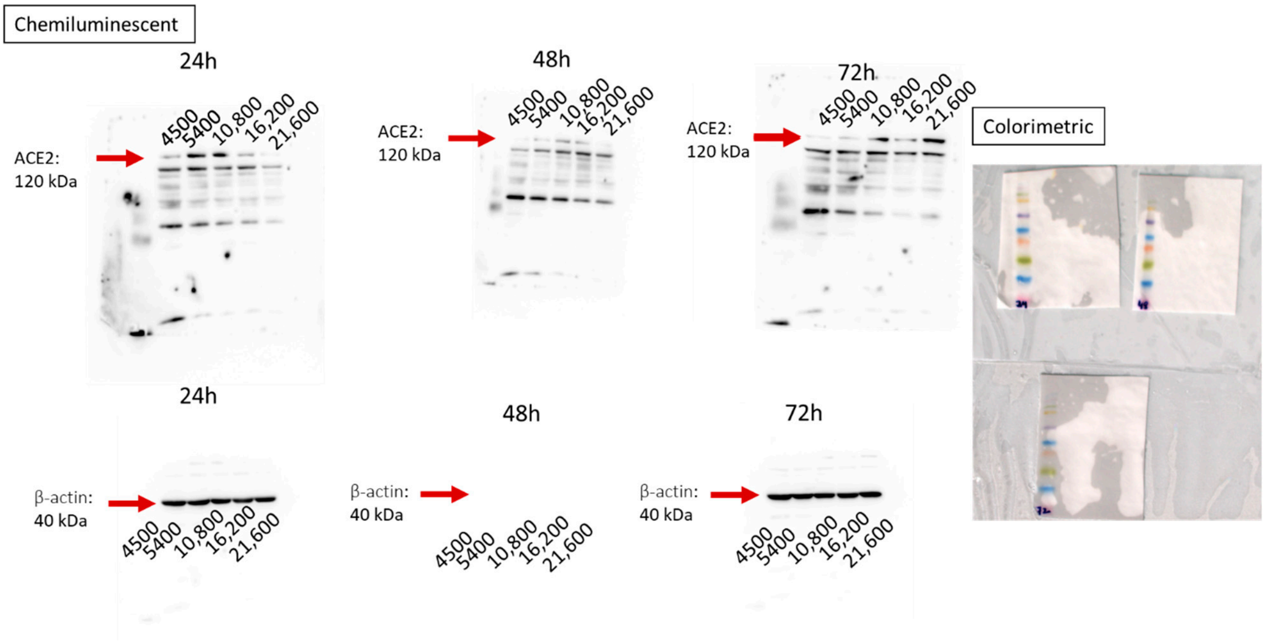

Figure S2

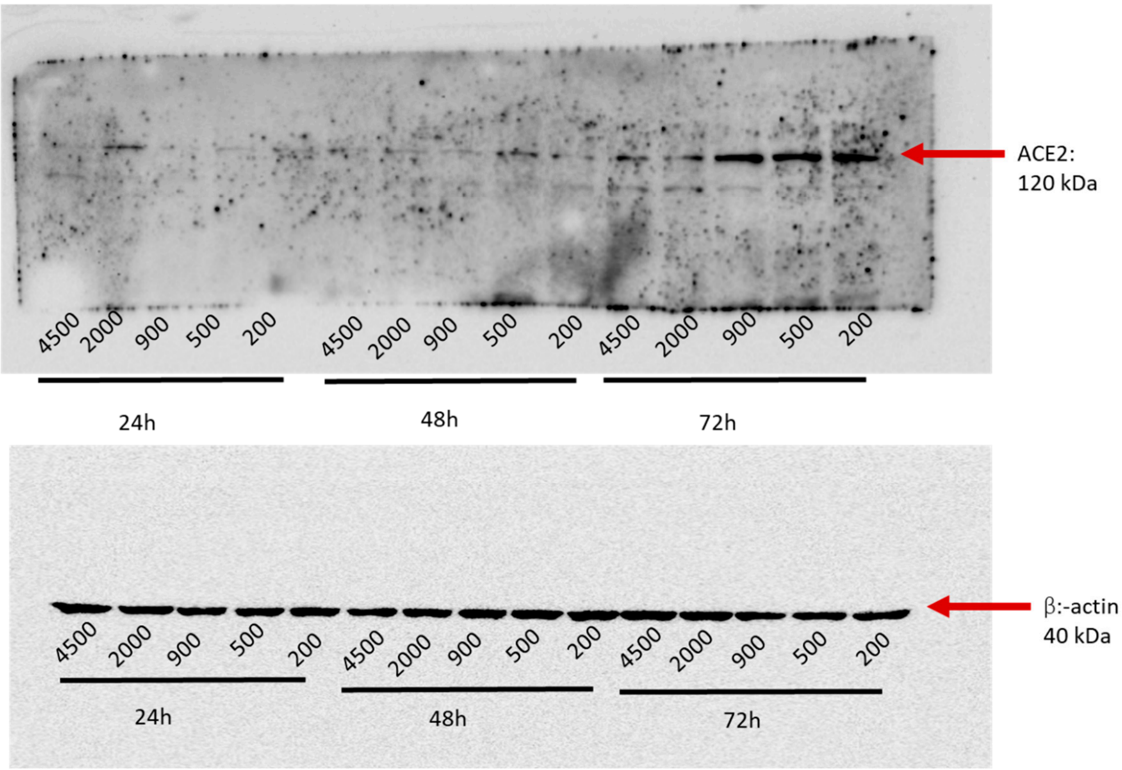

Figure S3

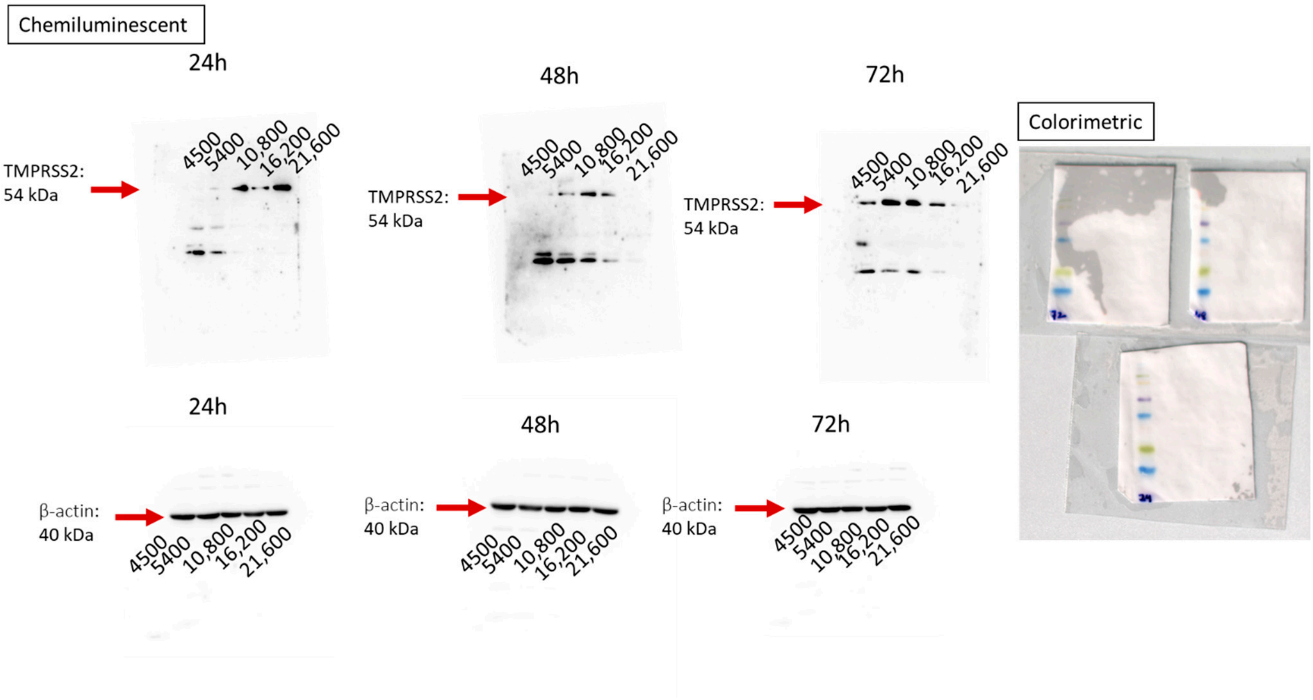

Figure S4

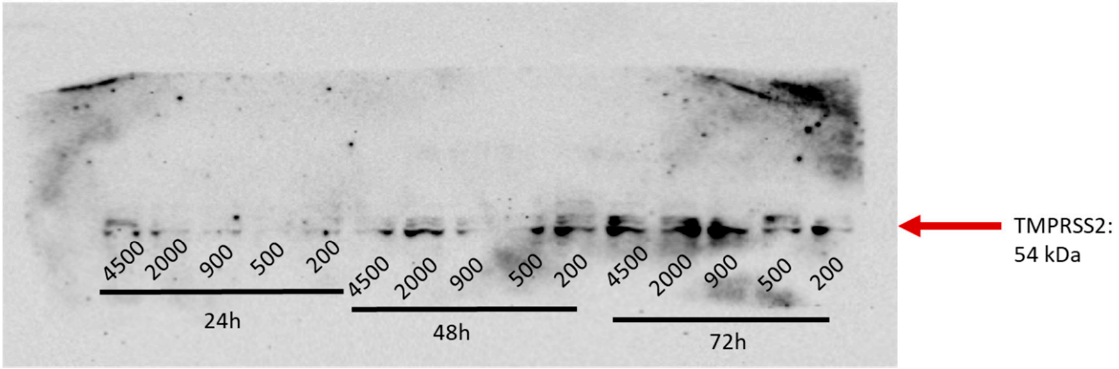
4500
2000
900
500
200
24h
4500
2000
900
500
200
48h
4500
2000
900
500
200
72h
TMPRSS2:
54 kDa

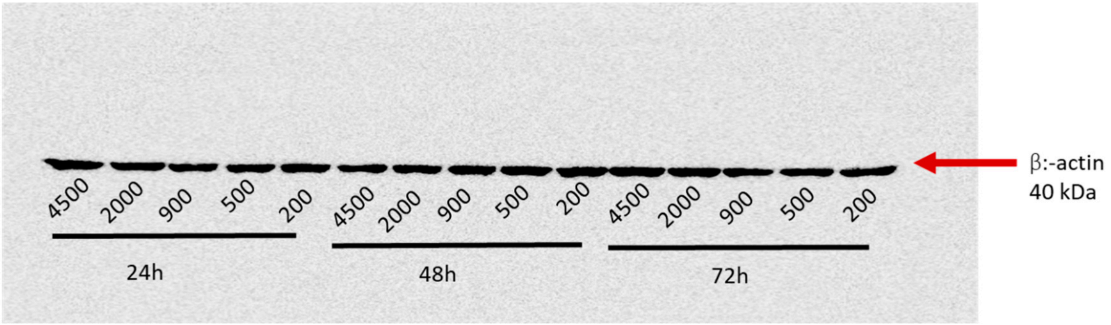
4500
2000
900
500
200
24h
4500
2000
900
500
200
48h
4500
2000
900
500
200
72h
β:-actin
40 kDa
